# New Player on An Old Field; the Keap1/Nrf2 Pathway as a Target for Treatment of Type 2 Diabetes and Metabolic Syndrome

**DOI:** 10.2174/1573399811309020005

**Published:** 2013-03

**Authors:** Dionysios V Chartoumpekis, Thomas W Kensler

**Affiliations:** Department of Pharmacology and Chemical Biology, School of Medicine, University of Pittsburgh, Pittsburgh, PA, 15261, USA

**Keywords:** Diabetes, Keap1, Metabolism, Nrf2, obesity, Oxidative stress.

## Abstract

Nuclear erythroid factor 2 like 2 (Nrf2) has been described as a transcription factor that serves as a master regulator of the adaptive response to exogenous and endogenous oxidative and electrophilic stresses. Evidence of Nrf2 crosstalk with other molecular pathways is increasing; recent publications have proposed a role of Nrf2 in the development of obesity and in the highly regulated process of adipocyte differentiation through its interaction with other transcription factors and receptors implicated in metabolic regulation. In the present review, we discuss the available data on the possible role of Nrf2 in obesity and metabolic syndrome and the feasibility of using Nrf2 as a therapeutic target in the clinical setting.

## 1 INTRODUCTION

The present review aims to highlight the recent research progress that has been made in the field of the transcription factor Nrf2 (nuclear erythroid factor 2 like 2) with reference to its role in the development of type 2 diabetes, obesity and metabolic syndrome and its potential use as a therapeutic target.

Although the beneficial effects of a lifestyle change (increased physical activity and dietary changes), in people who are already overweight or obese have become widely known [1, 2], the prevalence of obesity, metabolic syndrome and type 2 diabetes (T2D) continue to be high according to the US Center for Disease Control and Prevention. Indeed, 34.2% of US adults 20 years and older are overweight, 33.8% are obese and 5.7% are extremely obese, based on the Body Mass Index (BMI) [3]. The percentage of adults with T2D is about 11.3% (both diagnosed and estimated undiagnosed) [4]. Metabolic syndrome which usually comprises clinical findings such as insulin resistance, dyslipidemia (high triglyceride and low high-density lipoprotein (HDL) levels), central obesity and impaired glucose tolerance or T2D [5], has also become a major health concern as it affects more than 50% of the elderly in the USA [6] and increases the risk of atherosclerosis and related cardiovascular events.

Several lines of treatment options for obesity, T2D and metabolic syndrome are now available. The anti-obesity drugs mainly act by suppressing appetite or by preventing fat absorption and are prescribed along with advice on lifestyle. Two of them were recently approved by the US Food and Drug Administration (FDA); lorcaserin [7] and phentermine/topiramate [8]. 

A variety of treatment options for T2D prior to the recourse of exogenous insulin administration is available. Metformin is usually the first treatment of choice, and leads to decreased hepatic glucose production and increased peripheral glucose uptake and utilization [9]. Other drugs such as sulfonylureas and meglitinides stimulate pancreatic insulin secretion [10]. Thiazolidinediones are peroxisome proliferator-activated receptor gamma (PPARγ) agonists and improve insulin sensitivity [11] although the circulation of some of them has been suspended in Europe due to concerns about cardiovascular safety [12] and increases in bladder cancer incidence [13]. Dipeptidyl-peptidase 4 inhibitors increase the glucagon-like peptide-1 (GLP-1) levels and GLP-1 agonists have beneficial effects on increasing insulin secretion and decreasing glucagon secretion [14]. Alpha-glucosidase inhibitors act by slowing the digestion of complex carbohydrates [15]. Last but not least, the use of statins (3-hydroxy-3-methyl-glutaryl-CoA reductase inhibitors) in the treatment of dyslipidemia in metabolic syndrome has proved that their beneficial effects in lowering the cardiovascular risk can not be attributed to their LDL cholesterol lowering effects [16] only, but also to other pleiotropic (antioxidant, anti-inflammatory) actions of statins [17]. 

Despite the availability of several drug options for T2D and metabolic syndrome, the research for the identification of novel targets for these diseases is ongoing as no medication is considered a panacea. Novel treatment interventions that may be used as a monotherapy or along with existing therapies are needed. Based on this notion, the research community became attracted by the association between metabolic syndrome and oxidative stress [18-20]. Oxidative stress that is defined as an imbalance between the oxidant molecules (such as Reactive Oxygen Species-ROS) and the cell’s antioxidant defense mechanisms has been implicated in the development of insulin resistance. Houstis *et al.* provided the first solid mechanistic evidence that ROS may have a causative role in insulin resistance development [21]. Hence, the manipulation of molecular pathways that produce or eliminate ROS fluxes is becoming a popular means for potential intervention for metabolic syndrome treatment. In this context, NF-E2-related factor 2 like 2 (Nrf2) came under the spotlight in obesity research as it is a transcription factor that regulates the adaptive response to endogenous and exogenous oxidative or electrophilic stresses [22].

**Fig. (1). Brief schematic presentation of the Keap1/Nrf2 system.**
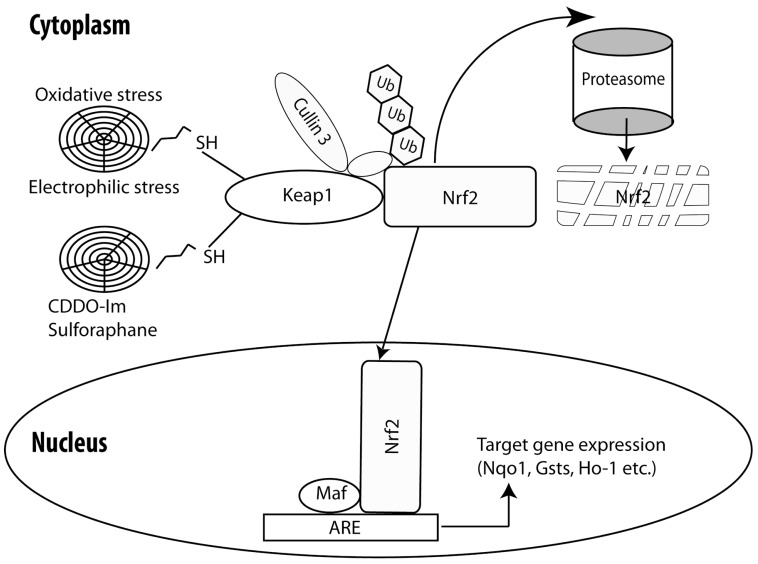
Nrf2 is bound to Keap1 in the cytoplasm. Keap1 acts as a substrate adaptor protein for the cullin 3-containing E3-ligase complex and targets Nrf2 for ubuquination. Then, Nrf2 is led to proteasome mediated degradation. Electrophilic and oxidative stressors or pharmacological inducers of Nrf2 signaling (CDDO-Im and sulforaphane) interact with cysteine thiols of Keap1to disrupt Nrf2 sequestration and proteolytic marking. Nrf2 can now accumulate, translocate into the nucleus and form heterodimers with the small Maf proteins, bind to Antioxidant Response Element (ARE) sequences in the regulatory domain of its target genes and activate their transcription. Nrf2; nuclear erythroid factor 2 like 2, Keap1; kelch-like ECH-associated protein 1, CDDO-Im; 1-[2-cyano-3,12-dioxooleana-1,9(11)-dien- 28-oyl]imidazole, Ub;ubiquitin, Maf; avian musculoaponeurotic fibrosarcoma AS42 oncogene homolog, Nqo1; NAD(P)H dehydrogenase, quinone 1, Gst;glutathione S-transferase, Ho-1; heme oxygenase-1.

Nrf2 belongs to the family of the cap n’ collar transcription factors with a basic leucine zipper. The invertebrate homologs of Nrf2, SKN-1 (*Caenorhabditis elegans*) and CncC (*Drosophila melanogaster*) also play an important role in mediating a cytoprotective response against stressors [23, 24]. Nrf2 is bound to Kelch-like ECH-associated protein 1 (Keap1) in the cytoplasm, which facilitates its proteasomal degradation [25]. Extrinsic or intrinsic stimuli (electrophilic and oxidative stress, chemical inducers such as triterpenoids, dithiolethiones, isothiocyanates) can modify reactive cysteines in Keap1 [26] and cause dissociation of Nrf2 from the Keap1/Nrf2 complex or conformational changes in Keap1 that rescue Nrf2 from proteasomal degradation. Consequently, Nrf2 can now accumulate, translocate to the nucleus and bind to Antioxidant Response Element sequences (ARE; 5’-NTGAG/CNNNGC-3’) in the regulatory domains of the Nrf2 target genes, thereby activating their transcription (Fig. **1**). Such cytoprotective Nrf2-induced genes are involved in conjugation/detoxication reactions (e.g. glutathione S-transferases-Gsts),antioxidative responses (e.g. NADPH quinone oxidoreductase –Nqo1) and proteasome function (proteasome subunits) [27]. 

As Nrf2 is ubiquitously expressed, the implication of its roles in a variety of pathologies [28] has been described mainly through the use of Nrf2 knock-out mice (Nrf2-KO) in disease models [29, 30]. Nrf2-KO mice have been shown to have increased susceptibility to chemical-induced carcinogenesis [31, 32], pulmonary inflammation [33], neurodegenerative inflammatory diseases [34] and acetaminophen hepatotoxicity [35]. These pathological conditions had already been associated with increased oxidative stress and researchers made the plausible hypothesis that Nrf2, being a central orchestrator of antioxidant gene expression, may participate in the pathophysiological mechanisms of these diseases. 

However, the evidence that the Keap1/Nrf2 pathway also cross-talks with other molecular pathways and transcription factors is growing [36]. Consequently, the role of Nrf2 in biological systems/pathological conditions may have more aspects than its mere antioxidant effects. A cogent example of the potential multifaceted effects of Nrf2 is its emerging role in metabolic syndrome and T2D. In this review we will discuss the recent research results on the role of the Keap1/Nrf2 pathway in metabolic regulation and suggest the potential manipulation of this pathway for the prevention and treatment of metabolic syndrome. 

## 2 ROLE OF NRF2 DELETION (LOSS OF FUNCTION) IN HIGH-FAT DIET-INDUCED OBESITY

The most widely used model of high-fat diet (HFD)-induced obesity is the C57BL6J mice under a regimen that provides 60 kcal% fat [37]. Studies on the role of Nrf2 in development of HFD-induced obesity and T2D have been performed using Nrf2-KO mice in this genetic background or wild-type (WT) mice treated with inducers of Nrf2 signaling. As increased oxidative stress has been described in HFD-induced obesity and it has been proposed that it may be associated with the accompanying insulin resistance, a plausible hypothesis would be that deletion of Nrf2 leads to increased oxidative stress in tissues (e.g. liver, adipose tissue, muscle) and exacerbates the resulting metabolic phenotype. Surprisingly though, the data from these studies do not support this hypothesis.

In 2008 Tanaka *et al.* employed a short-term HFD in WT and Nrf2-KO mice with duration of 1 or 4 weeks [38] in which they focused their study on liver. During this period no difference in weight gain was noticed between the different genotypes. Mouse livers with Nrf2 deletion were found to have higher mRNA expression levels of cholesterol uptake and synthesis genes, such as scavenger-receptor class B type 1 (SR-B1), low density lipoprotein receptor (LDLR), 3-Hydroxy-3-Methyl-glutaryl (HMG)-CoA synthase and HMG-CoA reductase, than the WT counterparts on the same diet. This study did not reveal if Nrf2 signaling is implicated in obesity as it was short-term and the mice had not become markedly obese, but it described that in the initial days of HFD feeding Nrf2 is down-regulated and that complete absence of Nrf2 increased the expression of lipid and cholesterol synthesis genes.

A longer-term (3-month) HFD (41kcal% fat) feeding study by Pi *et al.* showed Nrf2-KO mice gain less weight than the WT [39]. This study focused on white adipose tissue (WAT) and described that Nrf2-KO mice have lower amounts of WAT and the white adipocytes are also smaller than in WT mice. This difference in weight gain could not be attributed to differences in chow consumption, excretion of triglycerides or differences in activity but it could, at least partially, be explained by decreased adipogenesis in the Nrf2-KO mice as the authors described for the first time that Nrf2 can activate the promoter activity of PPARγ, a transcription factor with a central role in the expression of the adipogenic program [40, 41]. Although this study did not assess whether there are any differences in the insulin sensitivity between the two genotypes after HFD, it provided evidence that Nrf2 can directly regulate the expression of another transcription factor with a central role in metabolism and have an effect on the resulting phenotype (obesity).

A much longer HFD feeding (60 kcal% fat) (6 months) also revealed that Nrf2-KO mice tend to gain significantly less weight than the WT over time and that they are more insulin sensitive and more glucose tolerant after HFD feeding [42]. The increased circulating Fibroblast Growth Factor 21 (FGF21) levels of the Nrf2-KO mice after HFD feeding can contribute to their more insulin sensitive phenotype. FGF21 has relatively recently been described to act as a hormone and protect from obesity, increase fat utilization and energy expenditure and improve insulin sensitivity [43-46]. The increased FGF21 plasma levels in Nrf2-KO mice are observed only after HFD and parallels increases in Fgf21 mRNA levels in two of its major sources, liver and WAT. Under the long-term metabolic stress of HFD and also under the resulting increased oxidative stress, the Nrf2 levels in liver and WAT are increased in WT mice and can potentially repress FGF21 expression. This repression is abrogated in Nrf2-KO mice and Fgf21 is allowed to increase to a greater degree and exert its beneficial metabolic effects. mRNA levels of peroxisome proliferator-activated receptor gamma coactivator 1-alpha (PGC-1α), a gluconeogenesis, fatty acid oxidation and mitochondrial biogenesis coordinator [47], and phosphoenolpyruvate carboxykinase (PEPCK),a key gluconeogenic enzyme, which are FGF21-regulated genes, were also found to be increased in liver and WAT of Nrf2-KO mice. Although these data suggest that the Nrf2-KO mice under HFD have an ameliorated metabolic transcriptional program which can partially explain their phenotype, it is not evident which tissue plays the major role in this phenomenon and no information on energy expenditure and respiratory exchange ratio is available from this study. 

A first approach to the question about which tissue is principally implicated in the protection of Nrf2-KO mice from obesity and insulin resistance was made by Meher *et al.* [48]. This group showed that Nrf2 deficiency in myeloid cells is not sufficient to protect mice from adipose tissue inflammation and insulin resistance by using a model of bone marrow transplantation. Expanding on this kind of approach, it would be useful to make use of the Cre-loxP system [49] and directly target Nrf2 signaling in the tissue of interest (e.g. liver, adipose tissue, muscle, pancreas). This approach will help to avoid interference of endo- or paracrine effects from other tissues with Nrf2 deletion (as it may happen in the whole body Nrf2-KO mouse) to the tissue of interest. In this way it will become feasible to distinguish the effects of Nrf2 signaling in a specific tissue in an *in vivo *model (mouse) under basal (standard diet) and ‘metabolic stress’ conditions (HFD).

## 3 ROLE OF NRF2 ACTIVATION (GAIN OF FUNCTION) IN HIGH-FAT DIET-INDUCED OBESITY

Another approach to study the role of Nrf2 in HFD-induced obesity and T2D is the use of pharmacological substances that activate the Keap1/Nrf2 pathway by interacting with the reactive cysteines in Keap1 and accelerate nuclear accumulation of Nrf2. The synthetic triterpenoid analog of oleanolic acid 1-2-cyano-3,12-dioxooleana-1,9(11)-dien-28-oylimidazole (CDDO-Im) has been described as a potent inducer of Nrf2 signaling *in vivo* and *in vitro *[50]. A report by Shin *et al.* demonstrated that CDDO-Im treatment can protect WT mice from a long-term (3 months) HFD (60kcal% fat) -induced obesity [51]. The data suggest that CDDO-Im treated mice have increased energy expenditure and higher oxygen consumption; however no data are available on the insulin sensitivity or the tissue(s) responsible for this increased energy expenditure. Decreased levels of fatty acid synthase and acetyl-CoA carboxylase 1 in livers of CDDO-Im treated mice on HFD suggest a down-regulation of fatty acid synthesis while a decrease in acetyl-CoA carboxylase 2 indicates increased β-oxidation. The differential expression of these genes can explain the decreased lipid accumulation in the livers of CDDO-Im treated mice on HFD. It is also noteworthy that in this study Nrf2-KO mice were also used; the Nrf2-KO mice as shown in previous studies with Nrf2 deletion gained less weight than their WT counterparts on HFD but no further protection was induced by treatment with CDDO-Im. Consequently, this study indicated that Nrf2 signaling activation may also protect mice from a HFD-induced obese phenotype by increasing their energy expenditure. However, conclusions should not be drawn by direct comparisons of the results with the other studies using Nrf2-KO mice as this study used female mice while the others used male; as has been previously described, the presence of high estrogens concentration in the female mice may attenuate at a degree the effects of HFD-induced obesity [52].

In another study, Yu *et al.* described that continuous treatment of mice with oltipraz, another small molecule activator of Nrf2, protected them from HFD-induced obesity and insulin resistance [53]. This group mainly described an amelioration in the insulin signaling in white adipose tissue in the treated mice. 

Both these studies on Nrf2 activation and its effects in diet-induced obesity converge in the endpoint which is the protection from an obese and insulin resistant phenotype. However, inasmuch as these studies are using pharmacological interventions, it is possible that these agents may also have non-Nrf2 related effects. The use of Nrf2-KO mice in the study by Shin *et al.* [51] was helpful to rule out such off-target effects. Another approach could be the use of mice with genetic activation of Nrf2 signaling by manipulation of Keap1 [54]. A very recent study by Xu *et al.* [55] used Keap1 flox/flox mice that are actually Keap1 hypomorphs that serve as a genetic model of gain of Nrf2 function. In this study the authors also made use of a genetic model of obesity, the Lep^ob/ob^ mouse that is homozygous for a mutation in the leptin gene and become obese, hyperphagic, hyperglycemic and glucose intolerant and produced the Keap1 flox/flox:Lep^ob/ob ^mouse. They showed that Nrf2 gain of function in the Lep^ob/ob ^background attenuated lipid accumulation in WAT, exacerbated insulin resistance and did not have any effect on the weight again. In the same study, a short-term (5 weeks) HFD-induced obesity experiment was performed on wild-type and Keap1 flox/flox mice where it was shown that Nrf2 gain of function protected mice from obesity and led to less WAT accumulation. However, no data are available about the insulin sensitivity of these mice. Therefore, extension of the study on the HFD-induced obesity model using Keap1 flox/flox mice would be helpful to better assess the protective effects of genetic Nrf2 activation in the development of obesity as it is a more physiological model of obesity compared to the aforementioned genetic one.

## 4 NRF2 CROSSTALK WITH METABOLIC PATHWAYS

**Fig. (2). Summary of known Nrf2 crosstalk with pathways of metabolic interest.**
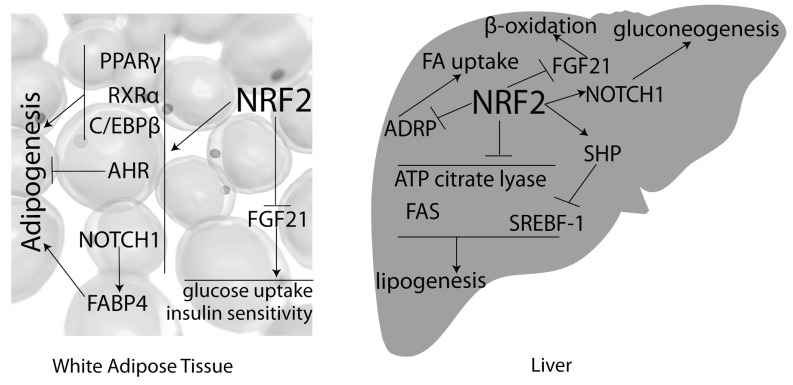
Most of the studies on Nrf2 and obesity-metabolic syndrome have focused on liver and white adipose tissue. Nrf2 has been shown to induce metabolic regulators (such as PPARγ, C/EBPβ, AHR in adipose tissue and SHP in the liver) or to repress other ones (such as FGF21, ATP citrate lyase). The exact molecular mechanisms of the majority of these interactions as well as their physiological significance are not clear yet. **→**denotes induction, --**|** denotes repression. Nrf2; nuclear erythroid factor 2 like 2, PPARγ;peroxisome proliferator-activated receptor gamma, RXRα;retinoid X receptor alpha, C/EBPβ; CCAAT/enhancer-binding protein beta, AHR;aryl hydrocarbon receptor, FGF21;fibroblast growth factor 21, FABP4; fatty acid binding protein 4, SREBF-1;sterol regulatory element binding transcription factor 1,FAS;fatty acid synthase, ADRP;adipose differentiation-related protein, SHP;small heterodimer partner, FA;fatty acid.

Implication of Nrf2 in the development of obesity and metabolic syndrome is based on the interaction of Nrf2 with other transcription factors or with the regulation of the expression of molecules (e.g. enzymes) that play a role in metabolism. The studies on HFD-induced obesity and Nrf2 that we described have already given a hint of such interactions. (Fig. **2**) summarizes known Nrf2 cross-talk with pathways of potential metabolic relevance.

### 4.1 Nrf2 and Adipogenic Pathways 

Nrf2 deletion has been shown to impair adipogenesis through PPARγ [39]. PPARγ is a master regulator of adipocyte differentiation and its interaction with Nrf2 provides an interesting link between Nrf2 and adipogenesis. Another study from the same group described that Nrf2 may also regulate the transcription of CCAAT/enhancer-binding protein β (C/EBPβ) [56] that is expressed early during adipogenesis and contributes to the initiation of the expression of PPARγ [57]. However, the exact role of Nrf2 in adipocyte differentiation has not been fully elucidated and there are phenomenically contradicting reports; another study has shown that Nrf2 induces aryl-hydrocarbon receptor (AHR) [58] which in turn can inhibit adipogenesis [59]. Moreover, treatment of cell lines with Nrf2 activators such as sulforaphane [60] and CDDO-Im [58] inhibited adipocyte differentiation. These discrepancies in the currently available data on Nrf2 and adipocyte differentiation may be due to the variety of the different cellular models that have been used and possibly because of differences in the levels of ROS flux during these experiments. Specifically, manipulation of Nrf2 expression may have an effect on the glutathione potential and consequently on ROS levels. ROS have been show to also act as signaling molecules [61] and to facilitate adipogenesis by accelerating mitotic clonal expansion and increasing C/EBPβ transcriptional activity [62]. The transcriptional activity of Nrf2 itself has been reported to decline during adipocyte differentiation using as a model the ST2 cell line [63]. Therefore, there might be a fine balance between the effects Nrf2 exerts through its interaction with other transcription factors and through inducing antioxidant pathways. The study that described impairment in adipogenesis in both sulforaphane treated and Nrf2 knock down 3T3-L1 preadipocyte cell line, attributed this phenomenon to the effect Nrf2 has on retinoid X receptor alpha (RXRα) [60]; RXRα was described to be induced by Nrf2 and its induction by Nrf2 activation or repression by Nrf2 knock-down impairs the formation of PPARγ/ RXRα heterodimers that are critical regulators of adipogenesis [64]. Moreover, the recently described association of the Keap1/Nrf2 pathway with the Notch homolog 1 (Notch1) signaling in regenerating liver [65] indicates that Nrf2 may also potentially affect adipogenesis through Notch1; Notch1 has been shown to be implicated in adipogenesis by controlling the expression of fatty acid binding protein 4 (FABP4) [66, 67].

The use of histological sections and of primary adipocyte cultures from mice with specific deletion of Nrf2 in adipose tissue could shed light into the exact role of Nrf2 in adipogenesis in an *in vivo* model without the interference of indirect effects by other Nrf2-KO tissues, as it happens in the whole body Nrf2-KO model. 

### 4.2 Nrf2 and Lipid Metabolism Pathways

The implication of Nrf2 signaling as a modifier of lipid metabolism was first addressed in HFD-induced obesity studies. It is known that the increased prevalence of obesity and T2D is associated with increased cases of non-alcoholic fatty liver disease (NAFLD), which is possibly the result of insulin resistance and impairment in lipid homeostasis (lipid synthesis, catabolism and secretion) [68]. The group that employed a short-term (1-month) HFD feeding in WT and Nrf2-KO mice [38] reported the initial response of liver to the HFD before the onset of obesity and insulin resistance. They showed that Nrf2-KO mice had increased hepatic free fatty acids after HFD than their WT counterparts, which is consistent with the higher mRNA levels of lipid synthesis (fatty acid synthase-FAS) and intracellular transport genes (fatty acid binding protein 1-FABP1) in Nrf2-KO mice. Accordingly, a longer-term HFD treatment with the Nrf2 activator CDDO-Im led to decreased hepatic levels of FAS [51]. On the other hand, Huang *et al.* using a 3-month HFD (42 kcal% fat) regimen showed that Nrf2 can positively regulate the orphan receptor small heterodimer partner (SHP) [69]. This results in increased hepatic lipid export and decreased lipid import in the liver in the Nrf2-KO mice after the 3-month HFD feeding period. However, it is noteworthy that this is the only study that showed Nrf2-KO mice to gain more weight than the WT, something that should be clarified and can possibly be due to an inadequate backcrossing to the C57BL6J background. Therefore, it seems that the response of the Nrf2-KO liver to HFD differs between the initial stages of feeding and longer-term feeding. This dichotomy indicates that as soon as increased oxidative stress and development of obesity and insulin resistance occur after long-term HFD, the role of Nrf2 in lipid handling may be modified possibly by interacting with other transcription factors through processes that remain to be elucidated. 

Other studies on lipid metabolism and Nrf2 used the methionine- and choline-deficient diet (MCD) as a model of fatty liver. This diet type does not induce insulin resistance as HFD and consequently it is not a model of T2D [70]. The mechanisms underlying the MCD-induced fatty liver after just weeks on the diet involve the impairment of very low density lipoprotein (VLDL) secretion because of the lack of phosphatidyl-choline synthesis [71] and the increased uptake of fatty acids [72]. The use of MCD as a model of NAFLD has revealed that Nrf2 deletion accelerates the onset of NAFLD and its progression to steatohepatitis [73, 74] while Nrf2 activation attenuates the progression of NAFLD [75]. Chowdhry *et al.* decribed that Nrf2-KO mice under MCD have increased levels of adipose differentiation-related protein (ADRP), that promotes accumulation of triacylglycerols and stimulates the uptake of fatty acids, and increased levels of cytochromes P450 involved in the metabolism of fatty acids (Cyp2e1 and Cyp4a10) [73]. Zhang *et al.* reported increased levels of CD36 (a fatty acid transporter) and FGF21 in the livers of Nrf2-KO fed a MCD [75]. FGF21, as presented earlier in this review, was also found elevated in HFD-fed Nrf2-KO mice [42]. 

Liver mRNA profiling of mice with genetic and pharmacological activation of Keap1/Nrf2 signaling [76] and proteomic analysis in livers from Nrf2-KO mice [77] have provided information about the effect of Nrf2 on lipid metabolism. Yates *et al.* described that activation of Nrf2 signaling in murine liver on a standard diet represses the expression of a series of enzymes involved in fatty acids biosynthesis and uptake, such as sterol regulatory element-binding factor-1 (SREBF-1), FAS, ADRP and ATP citrate lyase, that can at least partially explain the lower liver lipid levels [76]. Kitteringham *et al.* using a proteomics-based study, showed deletion of Nrf2 in mouse liver under standard diet upregulated proteins that are involved in lipid metabolism and mainly lipogenesis [77]. ATP citrate lyase was among the highest upregulated ones. Taken together, these data suggest that Nrf2 may have a repressive role on genes involved in lipid metabolism. Detailed studies on the molecular mechanisms of this regulation are warranted so as to establish this role. Furthermore, studies should be performed both under standard and high-fat diet for 3-month periods or longer in order to establish obesity and T2D and allow firmer conclusions for the role of Nrf2 in lipid metabolism under these metabolic conditions.

### 4.3 Nrf2 in Insulin Signaling Pathway and Glucose Handling

*In vivo *studies have shown that under basal conditions (standard diet) no difference is observed in the glucose levels and the insulin sensitivity between WT and Nrf2-KO mice [42]. After long-term HFD the Nrf2-KO mice are relatively protected from obesity and insulin resistance and glucose intolerance compared to their WT counterparts, as was presented earlier [42, 48]. Although these data demonstrate what is happening at the level of the organism (mouse), information about the effect of Nrf2 on insulin signaling in certain tissues (liver, muscle, adipose tissue) is scarce. A more in-depth analysis of insulin sensitivity in Nrf2-KO mice is needed using the gold-standard method of the hyperinsulinemic-euglycemic clamp [78, 79] and combining it with the use of radioactively labeled glucose or insulin so as to be able to measure insulin-stimulated glucose transport activity in separate tissues [79]. 

Further *in vitro *studies that describe the role of manipulation of the Nrf2 pathway to insulin signaling (Insulin receptor substrate 1 phosphorylation-IRS-1, PI3K/Akt) are not known. A group used the hepatocellular carcinoma HepG2 cell line and showed that Nrf2 knockdown by siRNA led to impaired insulin-stimulated Akt phosphorylation [53]. Unfortunately, it is not safe to draw conclusions for insulin signaling and metabolism using cancerous cell lines as although cancer and normal cells use the same metabolic pathways, cancer cells are not so dependent on external signaling by insulin and other growth factors [80]. For similar reasoning, it is not safe to draw conclusions for normal metabolism from a recent study by Mitsuishi *et al.* showing that Nrf2 redirects glucose and glutamine into anabolic pathways [81], although this study has surely a big impact on the elucidation of mechanisms of metabolic reprogramming in cancer cells. A safer way we suggest to study the role of Nrf2 in insulin resistance *in vitro *is the use of primary cultures of hepatocytes from WT and Nrf2-KO mice and induction of insulin resistance with treatment with tumor necrosis factor-α or dexamethasone as described previously [21] followed by estimation of insulin signaling between the different genotypes.

In parallel, there is evidence that Nrf2 transcriptional activity can be affected by insulin signaling. Specifically, the glycogen synthase kinase-3β (GSK-3β), which is inactivated by phosphorylation by Akt after insulin receptor activation, can inhibit Nrf2 activity by phosphorylating Nrf2 and causing its nuclear exclusion and degradation [82, 83]. Hence, this interplay that seems to exist between Nrf2 and insulin signaling deserves further investigation.

## 5 CONCLUSION-FUTURE DIRECTIONS

Interest in the role of Nrf2 in metabolic syndrome and type 2 diabetes is growing. Three recently published reviews have also focused on the potential of the Keap1/Nrf2 pathway to serve as a target for these diseases. Yu *et al.* have mainly elaborated on the role of Nrf2 in insulin signaling and the possible association with oxidative stress [84]. Vomhof-DeKrey *et al.* mainly focused on the possibility that Nrf2 can be manipulated by nutrients such as fatty acids and thus exert its actions as a regulator of energy metabolism regulator [85]. Sykiotis *et al.* described the possible implication of Nrf2 in insulin signaling, lipid metabolism and its potential effects on longevity [86]. 

The majority of the studies on the role of Nrf2 have been performed using the Nrf2-KO mouse as a model. The existing data converge to the fact that Nrf2-KO mice are at least partially protected from obesity and insulin resistance when challenged with a long-term HFD. The mechanisms proposed include interaction of Nrf2 with other pathways (PPARγ, FGF21, lipid synthesis enzymes) mainly in liver and white adipose tissue. On the other hand, pharmacological activation of Nrf2 pathway has also been found to protect mice from obesity by increasing energy expenditure. Despite the phenomenical contradiction that both Nrf2 gain or loss of function may protect from obesity, this could happen through distinct mechanisms/pathways. Detailed assessment of insulin sensitivity and glucose tolerance of mouse models with Nrf2 deletion or activation within the same experiment will provide an answer to the question which manipulation of Nrf2 leads to a more insulin sensitive phenotype.

Moreover, as it is not clear which tissue exerts the principal role in the resulting metabolic phenotype after Nrf2 manipulation, it would be helpful to use genetic models with conditional deletion of Nrf2 (Nrf2 loss of function) or Keap1 (Nrf2 gain of function) in the major “metabolic” insulin sensitive tissues (liver, adipose tissue and muscle) using the Cre-loxP system. In this way it would be feasible to assess the role (if any) of each tissue to the resulting phenotype, excluding endo-or para-crine interactions, as it happens in the whole body Nrf2-KO mouse. 

Besides the elucidation of the exact mechanisms that underlie the effect of Nrf2 on obesity and metabolism in general, it is important to consider which options are already available for clinical manipulation of the Keap1/Nrf2 pathway. Total Nrf2 deletion or high Nrf2 activation that can be achieved with the genetic models may be helpful to understand mechanistically a phenomenon but are unlikely to be desirable at the clinical level. As it was stated in the introduction, total Nrf2 deletion increases susceptibility to carcinogens and other toxic insults. On the other hand Nrf2 activation is high in tumors that are chemoresistant and aids in the metabolic reprogramming that is necessary for the continuous replication of cancer cells. Therefore, a graded Keap1/Nrf2 signaling manipulation may be more preferable. On the one hand, the use of Nrf2 activators in obesity clinical trials could be an excellent option for graded Nrf2 activation. The synthetic triterpenoid bardoxolone methyl [87] or the naturally occurring isothiocyanate sulforaphane, in the form of extract from broccoli sprouts [88], are two examples of such activators that are currently being used in clinical trials. However, the early termination of a recent phase 3 clinical study (clinicaltrials.gov identifier NCT01351675) using bardoxolone methyl in patients with stage 4 chronic kidney disease and type 2 diabetes for the assessment of the efficacy of this triterpenoid in the protection against the progression to end-stage renal disease and the cardiovascular death, cautions that there is more to learn about the manipulation of this pathway. On the other hand, inhibitors of the Keap1/Nrf2 pathway are more limited [89], but in a recent review Magesh et al describe aspects of inhibiting the Nrf2 signaling such as Nrf2-based peptide inhibitor design [90]. At present, the Keap1/Nrf2 system appears to be an attractive target for obesity and metabolic syndrome treatment and prevention. As already described, some extra research steps are required so as to pinpoint the molecular mechanisms underlying the metabolic effects of Nrf2 signaling before proceeding to the proposition of relevant clinical trials.
